# Inclusive leadership and voice behavior among early-career nurses: the conditional roles of work meaningfulness and proactive personality

**DOI:** 10.3389/fpsyg.2026.1964826

**Published:** 2026-10-22

**Authors:** Xuerui Wang, Huiyu Wang, He Zhou

**Affiliations:** School of Economics and Management, East University of Heilongjiang, Harbin, China

**Keywords:** conditional indirect association, constructive voice, early-career nurses, inclusive leadership, proactive personality, work meaningfulness

## Abstract

**Introduction:**

Safety-focused explanations of nurse voice leave open whether the perceived value of work is associated with speaking up differently across proactive personality. Drawing on social information processing theory and an interactionist perspective informed by trait activation theory, we examined this conditional association among early-career nurses.

**Methods:**

A cross-sectional survey of 464 nurses in five tertiary hospitals in Harbin, China, assessed inclusive leadership, work meaningfulness, constructive voice, and proactive personality. Item-level confirmatory factor analysis and composite-score conditional process analysis were conducted.

**Results:**

Inclusive leadership was positively associated with meaningfulness and voice. The conditional indirect association through meaningfulness was positive at mean proactive personality (0.2502, 95% bootstrap CI [0.1857, 0.3197]) and increased with personality (index = 0.0744, 95% CI [0.0269, 0.1229]). The interaction added 1.29 percentage points of explained voice variance, indicating modest amplification; the personality–voice zero-order correlation was near zero.

**Discussion:**

The findings provide context-specific evidence for a conditional value-of-work account alongside safety-based explanations, without establishing causality or superiority over alternative pathways. Inviting suggestions and communicating the value of nursing contributions are practices worth evaluating. The data concern constructive voice, not demonstrated improvements in patient safety, and require longitudinal, multisource replication.

## 1 Introduction

Constructive voice involves voluntarily expressing suggestions and differing opinions to improve work practices (Van Dyne and LePine, 1998; Morrison, 2023). In nursing, hierarchical relationships can make such communication difficult, particularly when employees have limited experience or status (Krenz et al., 2020; Kee and de Jong, 2022). The present study concerns general improvement-oriented voice. It does not measure incident reporting, patient safety, or care quality, and these outcomes should not be inferred from the findings.

Inclusive leadership—leader openness, accessibility, and availability—offers a relevant social context for voice (Carmeli et al., 2010). Research links leader inclusiveness with psychological safety and improvement efforts in healthcare teams (Nembhard and Edmondson, 2006), and with nurses' voice and error reporting through psychological safety (Lee and Dahinten, 2021). Psychological safety concerns anticipated interpersonal consequences of expression. Work meaningfulness instead concerns whether work is experienced as significant and worth investing in (May et al., 2004). An employee may consider speaking up safe yet see little reason to invest effort in improvement, or value the work while still fearing adverse reactions.

Three questions motivate the study. First, a safety-centered explanation does not itself establish whether the perceived value of work is associated with constructive voice. Leadership–meaningful work–voice research already exists (Chen et al., 2018), so testing another positive indirect association alone would offer limited novelty. Second, the strength of the meaningfulness–voice association may differ with proactive personality, a tendency to initiate and persist in change (Bateman and Crant, 1993). Third, the applicability of this conditional pattern to nurses within their first 5 years of practice warrants examination, without assuming that it is unique to that career stage.

We therefore examine a specific conditional proposition: the positive indirect association between inclusive leadership and constructive voice through work meaningfulness is stronger at higher proactive personality because the meaningfulness–voice association is stronger. Social information processing theory motivates the leadership–meaningfulness association (Salancik and Pfeffer, 1978); an interactionist perspective informed by trait activation theory motivates the conditional association with voice (Tett and Burnett, 2003). This is a context-specific application and test of a proposed boundary condition, rather than evidence that either theory requires revision.

Early-career nurses provide a relevant setting because uncertainty about professional standing and the reception of suggestions may make leader responses salient (Kee and de Jong, 2022; Lee and Kwon, 2025). Career stage defines the population studied; it is not a tested moderator. With no experienced-nurse comparison group, the study cannot establish that leadership cues or the conditional indirect association are stronger in early-career nurses. The cross-sectional design further limits the findings to contemporaneous associations.

## 2 Theoretical background and hypotheses

### 2.1 Inclusive leadership, meaningfulness, and constructive voice

Social information processing theory holds that employees interpret work using cues from the surrounding social environment (Salancik and Pfeffer, 1978). When supervisors listen to suggestions and acknowledge contributions, nurses may interpret their work as valuable to the unit. This argument concerns the perceived significance of the work itself. It differs from psychological safety (anticipated interpersonal risk), respect (others' regard), belongingness (social inclusion), and organization-based self-esteem (personal worth within the organization). These appraisals may coexist. Meaningfulness is selected because it is proximal to the proposed value-of-work explanation, not because it has been shown to be the strongest or sole explanatory appraisal. None of the alternative appraisals was measured here.

Inclusive leadership may also be associated with voice through routes beyond meaningfulness. Approachable leaders may provide opportunities to contribute, foster expectations that suggestions will be considered, and reduce anticipated interpersonal costs (Detert and Burris, 2007; Lee and Dahinten, 2021). Retaining a direct leadership–voice coefficient allows for these omitted routes and avoids constraining the conditional direct association to zero. That coefficient cannot identify which alternative process operates.

H1: Inclusive leadership is positively associated with constructive voice behavior among early-career nurses.
H2: Inclusive leadership is positively associated with work meaningfulness among early-career nurses.

### 2.2 Meaningfulness and a reference-value indirect association

Perceiving work as significant provides a reason to invest effort in improving it. Related evidence links meaningful work with voice in a leadership model (Chen et al., 2018) and with performance in healthcare professionals (Zhou et al., 2023). These findings motivate the proposed association, but do not demonstrate the present conditional association. Meaningfulness may accompany voice because employees value improvement; voice and favorable work appraisals could also reinforce one another, or share unmeasured causes.

In the model below, the meaningfulness–voice coefficient depends on proactive personality. H3 and H4 therefore use the sample mean of proactive personality as an explicit reference. This wording aligns the hypotheses with the reported conditional tests; it does not redefine them as causal effects. The corresponding zero-order meaningfulness–voice correlation is reported separately.

H3: At the sample mean of proactive personality, work meaningfulness is positively associated with constructive voice, conditional on inclusive leadership and the other terms in the outcome equation.
H4: At the sample mean of proactive personality, inclusive leadership has a positive statistical indirect association with constructive voice through work meaningfulness.

### 2.3 Proactive personality as a boundary condition

Trait activation theory explains behavioral expression of personality in the presence of trait-relevant situational cues (Tett and Guterman, 2000; Tett and Burnett, 2003). Work meaningfulness is an individual psychological appraisal, not an objective situational cue. We therefore use this theory as an interactionist lens: perceiving an improvement goal as worthwhile may be associated with greater expression of an initiative-oriented disposition. The study does not directly measure situational cue strength or the activation process, so the interaction is not a direct test of trait activation.

The proposed location of moderation follows the substantive distinction between valuing work and acting on that valuation. Proactive personality may be particularly relevant to identifying opportunities and persisting in expressing suggestions once work is experienced as worthwhile. Prior work links proactive personality with voice through voice self-efficacy and contextual delegation (Xie et al., 2014). Xu et al. (2024) instead locate proactive personality at the leadership–work-reflection stage in a servant-leadership model. These studies support considering conditional relationships but do not establish the present second-stage specification or show that it is preferable to all alternatives.

The product term is mathematically symmetric: an interaction between meaningfulness and proactive personality can also be described as variation in the personality–voice slope across meaningfulness. Describing personality as the moderator organizes the focal leadership–meaningfulness–voice question; it does not establish an empirical direction for the interaction. Comparisons with models using measured contextual cues and alternative moderation locations require additional data and theory-specific tests.

A trait associated with initiative need not be identical to a specific act of constructive communication. Nevertheless, prior proactivity–voice research makes a positive zero-order relationship plausible (Xie et al., 2014). A weak or negative relationship would qualify a general personality explanation and requires substantive assessment; a significant interaction alone cannot resolve that discrepancy.

H5: The positive conditional association between work meaningfulness and constructive voice is stronger at higher proactive personality.
H6: The statistical indirect association between inclusive leadership and constructive voice through work meaningfulness increases with proactive personality, indicated by a positive index of moderated mediation.

H4 concerns the product of the first-stage coefficient and the meaningfulness–voice coefficient at mean proactive personality. H5 concerns the outcome-equation interaction. H6 concerns the product of the first-stage and interaction coefficients, which quantifies how the conditional indirect association changes per unit of proactive personality (Hayes, 2022). These are distinct statistical quantities; none establishes temporal ordering. Figure 1 summarizes the model.

**Figure 1:**
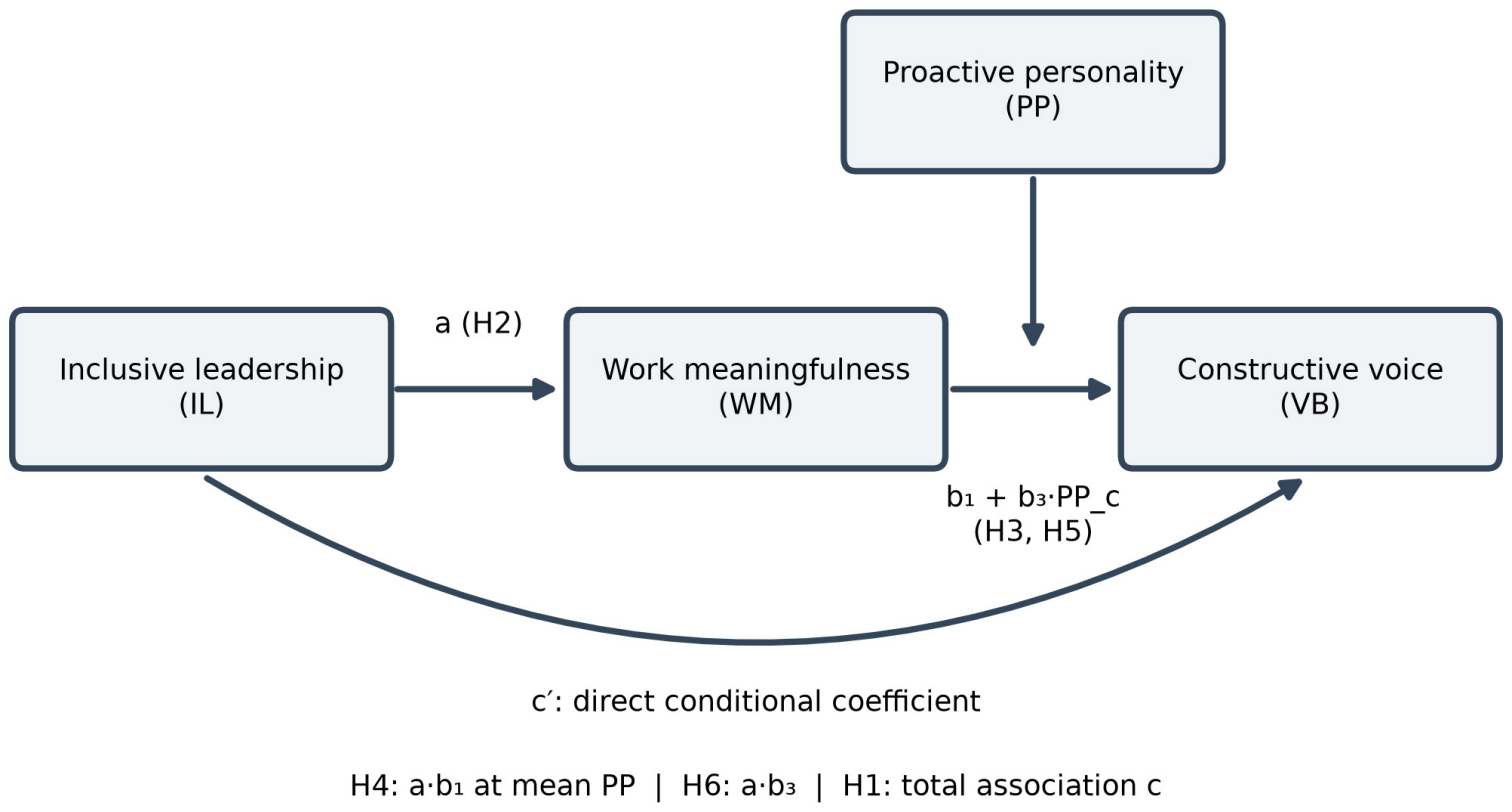
Proposed conditional association model. Arrows express the proposed statistical specification, not established temporal or causal ordering. H1 uses the separate total-association equation; c′ is the conditional direct coefficient. H3 and H4 use mean PP. H5 tests the interaction, and H6 tests its first-stage product.

## 3 Materials and methods

### 3.1 Design and participants

A cross-sectional questionnaire survey was conducted from 1 March to 31 May 2026 in five Grade A tertiary hospitals in Harbin, Heilongjiang Province, China. The authors report that the study protocol received prospective ethics approval before formal participant recruitment. Reporting was guided by the STROBE recommendations for cross-sectional observational research (von Elm et al., 2007).

Eligible participants were full-time registered nurses providing direct clinical care, within 5 cumulative years of full-time clinical practice, able to read Chinese, and able to rate an identifiable immediate supervisor. The five-year operational definition is consistent with the population described by Masamba et al. (2026). Electronic informed consent was required. Interns, unregistered trainees, students, exclusively non-clinical personnel, nurses with more than 5 years of clinical practice, and pilot participants were excluded.

### 3.2 Sampling, recruitment, and screening

The study sites were the First Affiliated Hospital of Harbin Medical University, the Second Affiliated Hospital of Harbin Medical University, Harbin Medical University Cancer Hospital, the Fourth Affiliated Hospital of Harbin Medical University, and the First Affiliated Hospital of Heilongjiang University of Chinese Medicine. These five Grade A tertiary hospitals were selected through accessible recruitment contacts and willingness to cooperate. Members of the human resources staff known personally to the investigator assisted with recruitment. They used personnel-record information to identify potentially eligible nurses and helped distribute the questionnaire. Both hospitals and participants were selected through non-probability procedures, with purposive identification of participants. The recruitment plan allocated 100 invitations per hospital, with targets of at least 470 submitted and 450 valid questionnaires. The total number of eligible nurses at each hospital was not counted. The 100-per-hospital figure was a recruitment allocation target, not an estimate of the eligible population or a population-proportional allocation. Individual inclusion probabilities were unknown.

Human resources staff sent the electronic questionnaire link individually through WeChat to eligible nurses identified from personnel records and registered invitation delivery. They knew which nurses had completed the questionnaire, and two reminders were directed to those who had not yet completed it. Nurses who agreed to fill in the questionnaire were told that they could collect Chinese New Year couplets from the human resources office. According to the investigator, the human resources staff did not view individual answers; this describes their reported practice and does not establish a technical restriction on access. Participation status was therefore known to the recruitment contacts, and the recruitment process was not anonymous. The first questionnaire page explained the study purpose, voluntary participation, confidentiality arrangements, and the right to withdraw without adverse consequences. The questionnaire itself did not request names, employee identification numbers, contact details, or other direct identifiers. The survey platform generated an IP-related duplicate-submission flag for screening; raw IP addresses were not included in the 464-case analytical dataset.

The investigator confirmed that questionnaire invitations were actually delivered to 500 people. The original study report records 476 submitted questionnaires. Twelve were excluded: three for clinical tenure above 5 years, five for more than 10% missingness among the 31 focal items, and four for the joint occurrence of an identical numeric category across all 31 items and completion in less than 60 s. The analysis comprised 464 retained questionnaires. The submission proportion relative to the reported delivered invitations was 95.2% (476/500), the retained-questionnaire proportion relative to delivered invitations was 92.8% (464/500), and the retained fraction of submissions was 97.5% (464/476). The delivered denominator is based on investigator confirmation; these recruitment-flow proportions are not conventional population response rates because no complete sampling frame was enumerated. They do not establish representativeness or equal participation opportunities.

The joint screening rule required both invariant numeric responding and very short duration; neither condition alone was an exclusion rule. Because the questionnaire combined five- and seven-point formats, choosing the same number across all items does not represent the same relative response position. This pattern was treated as a screening flag, not proof of invalidity. No record is available documenting the basis for selecting the 60-s cutoff or when it was determined, so its prespecification cannot be verified. In the retained sample, median completion time was 413 s (interquartile range 313.50–541.75; range 180–1200). These retained-sample times describe the data but do not independently validate the cutoff. The restricted analysis files contain only retained cases and do not permit independent reconstruction of the four reported joint-rule exclusions or a sensitivity analysis reinstating those cases.

No focal-item values were missing in the retained data, and all were integers within the specified response ranges. Scale scores were recomputed from all items rather than rounded saved means. The restricted dataset excludes direct identifiers and also excludes ResponseID, StartTime, EndTime, and IPDuplicateFlag. Consequently, recruitment delivery, exclusions, and platform duplicate checks cannot be reconstructed from this file alone. No two retained cases had identical full 31-item response vectors; this check is not a substitute for platform-level duplicate screening.

### 3.3 Sample-size rationale

The original planning calculation targeted the interaction in a regression with one tested and four total predictors: *f*^2^ = 0.05, two-sided α = 0.05, and power of 0.80, yielding approximately 159 participants using the *R*^2^*-*increase procedure in G^*^Power 3.1 (Faul et al., 2009; Shieh, 2009). The target of 450 valid cases provided a margin for the 31-item measurement model, bootstrap estimation, and data-quality screening. It was not derived from a model-specific CFA or conditional-indirect-effect power simulation. A non-central-F check of the same interaction assumptions gives power of approximately 0.997 at *N* = 450; this calculation does not account for clustering, measurement error, or a smaller interaction and does not validate the design retrospectively.

### 3.4 Translation and pilot assessment

Because the measures were originally developed in English, a forward–backward translation and cultural-adaptation procedure was used. The process emphasized conceptual rather than literal equivalence and included independent forward translation, synthesis, independent back-translation, expert review, and cognitive interviewing (Beaton et al., 2000; Brislin, 1970; Cha et al., 2007; Sousa and Rojjanasrirat, 2011).

Two researchers proficient in Chinese and English independently translated the items into Chinese. The research team then reconciled differences in wording, nursing terminology, and cultural expression to produce a preliminary Chinese version while preserving the conceptual content of each item.

A bilingual researcher who had not seen the original measures and had not participated in the forward translation independently back-translated the preliminary Chinese version. The research team compared the back-translation with the original measures and revised wording where semantic meaning, conceptual scope, or intensity differed (Brislin, 1970).

A five-member expert panel comprising two professors of nursing management, two senior clinical nursing directors, and one organizational behavior specialist reviewed the semantic, idiomatic, experiential, and conceptual equivalence of the translated measures. The panel also assessed the relevance, clarity, and cultural appropriateness of the items for early-career nurses (Beaton et al., 2000). Ambiguous wording was revised without changing the intended constructs or the positive scoring direction.

Cognitive interviews were then conducted with 15 eligible early-career nurses selected through purposive sampling, with three nurses recruited from each of the five participating hospitals. Using paraphrasing and verbal probes, participants explained how they interpreted each item and identified wording that was difficult to understand, ambiguous, or inconsistent with clinical nursing practice. This step supported response-process validity and helped identify problems that quantitative pilot testing alone might not reveal (Beatty and Willis, 2007). Feedback from all stages was integrated into the 31-item Chinese pilot questionnaire.

The Chinese-language questionnaire was distributed and pilot responses were collected from 8 to 30 January 2026, after ethics approval on 6 January 2026 and before the main survey. The pilot was conducted at the same five hospitals and yielded 158 valid responses. Human resources staff identified potentially eligible nurses using personnel records and distributed the pilot questionnaire link via WeChat. They recorded the nurses who had received pilot invitations and used these records to avoid inviting them again for the main survey. The pilot participants met the principal eligibility criteria for the main study. The investigator confirmed that no pilot participant was included in the formal sample. Pilot and formal datasets were analyzed separately, and pilot responses were not included in primary hypothesis testing. No item-level data were missing across the 31 study items. No questionnaire items, response options, or survey procedures were changed after the pilot.

Pilot reliability coefficients ranged from 0.847 to 0.905 (Table 1). KMO was 0.885, and Bartlett's test was χ^2^(465) = 2322.23, *p* < 0.001. Principal axis factoring used four factors specified from the instrument structure and Promax oblique rotation (power 4), because the constructs could correlate (Fabrigar et al., 1999). The reduced-matrix eigenvalues were 6.767, 5.008, 2.402, and 1.602, accounting for 50.90% of total item variance. The revision-stage reproduction yielded absolute primary loadings of 0.595–0.835 and maximum secondary loading of 0.237; no item was removed. Full pattern coefficients are in Supplementary File S4.

**Table 1:** Pilot reliability and reproduced exploratory factor analysis.

Construct	Items	Alpha	Primary loading range	Maximum secondary loading
IL	9	0.894	0.602–0.764	0.116
WM	6	0.847	0.595–0.826	0.151
VB	6	0.866	0.611–0.835	0.155
PP	10	0.905	0.618–0.780	0.237

*N* = 158. Principal axis factoring with Promax rotation (power 4); primary and secondary values are absolute pattern coefficients. KMO = 0.885; Bartlett χ^2^(465) = 2322.23, *p* < 0.001. Reduced-matrix factors account for 50.90% of total item variance. Revision-stage parallel analysis independently supports four factors; full results are in S4.

To assess rather than assume factor retention, a revision-stage parallel analysis compared correlation-matrix eigenvalues with the 95th percentiles from 2,000 independently column-permuted datasets, preserving each item's observed response distribution. The first four observed eigenvalues (7.248, 5.496, 2.882, 2.078) exceeded their reference values (2.079, 1.898, 1.781, 1.687), whereas the fifth (0.963) did not exceed 1.602. This supports four factors, but the retention check was added during revision and was not the original selection rule. Translation, pilot factor analysis, and the main-sample CFA provide preliminary measurement evidence, not comprehensive cross-cultural validation or evidence of invariance.

### 3.5 Measures and scoring

The 31 positively worded items measured inclusive leadership (IL; nine items, Carmeli et al., 2010), work meaningfulness (WM; six items, May et al., 2004), constructive voice (VB; six items, Van Dyne and LePine, 1998), and proactive personality (PP; ten-item short form, Seibert et al., 1999, based on Bateman and Crant, 1993). No reverse-scored item was specified in the supplied questionnaire and dictionary. Higher arithmetic item means indicate higher levels of each construct. Supplementary File S1 summarizes the actual measure sources, item counts, response formats, scoring, and adaptations; S2 documents variable coding. Complete item wording is omitted from the Supplementary File S1. The investigator confirmed that the Chinese items and response options in the originally submitted full questionnaire match those actually administered; that questionnaire is retained as the study record.

IL and WM used five-point agreement formats; VB and PP used seven-point agreement formats, each anchored from strongly disagree to strongly agree. The response formats were retained as specified in the study questionnaire rather than rescaled to a common range. IL refers to the immediate supervisor, WM to the significance of nursing work, VB to self-reported constructive suggestions and differing opinions, and PP to an initiative-oriented disposition. Raw means are interpreted within their own response formats, not compared as levels across constructs. VB does not directly measure incident reporting, safety outcomes, or objective improvement.

### 3.6 Ethics and confidentiality

The study was conducted in accordance with the Declaration of Helsinki (World Medical Association, 2024) and applicable Chinese requirements for ethical review of research involving human participants (National Health Commission of the People's Republic of China, 2023). The Ethics Committee of East University of Heilongjiang approved the protocol on 6 January 2026 (HLJDFXY20260106005), before pilot recruitment began on 8 January 2026. The pilot and main surveys were conducted under this protocol.

Before beginning the electronic questionnaire, eligible nurses received participant information describing the study purpose, procedures, expected burden, possible discomfort, confidentiality arrangements, data use, voluntary nature of participation, and right to withdraw before submission. Electronic informed consent was recorded before participation.

Participation was voluntary, and nurses could withdraw before questionnaire submission. Human resources recruitment staff knew individual participation status but, according to the investigator, did not view individual answers. The questionnaire did not request names, employee or national identification numbers, or contact details. Recruitment registration nevertheless involved identifiable WeChat contacts, so the absence of direct identifiers in the analysis file does not establish anonymity throughout data collection. Research outputs used aggregated results. Screening metadata and the de-identified analysis file serve different purposes; the restricted file used for this revision contains neither raw IP addresses nor the platform duplicate flag. The confidentiality procedures do not eliminate social-desirability or evaluation concerns (Standing Committee of the National People's Congress., 2021).

Electronic research data were stored on password-protected, encrypted devices or institutionally approved secure storage. The research data were used for the approved research purposes.

### 3.7 Statistical analysis

All revision estimates were reproduced locally from full-precision item means using Python 3.12.14, NumPy 2.5.3, pandas 3.0.5, SciPy 1.18.1, statsmodels 0.15.0, and semopy 2.3.11. The original report identified SPSS Statistics 29 and Amos 29; the revision supplies an independent, reproducible implementation rather than an unverified original software output. Tests were two-sided, with 95% confidence intervals and α = 0.05. Display values are rounded directly from full-precision estimates; supplementary measurement results retain additional decimal places. Regression coefficients and confidence limits use four decimals, and *t, F*, and *p*-values use three, with *p* < 0.001 for smaller values. Supplementary File S3 describes the implementation and accompanying executable code. The retained data were examined for ranges, missingness, response patterns, and influence; no further case was removed from the primary model.

CFA and composite-score regression had separate roles. CFA evaluated the item-level measurement structure. Each item loaded on one prespecified factor, all cross-loadings and residual covariances were fixed to zero, the four factors could correlate, and one loading per factor fixed the scale. The reproduced models used no modification-index-driven changes. Three alternatives merged WM with VB, then IL with WM and VB, or all four constructs. Maximum likelihood fitted the covariance structure treating item categories as continuous; Wishart (*N* – 1) scaling was used for χ^2^ and derived fit indices. SRMR is the root mean squared standardized off-diagonal covariance residual. The model syntax and full loadings are supplied in S3 and S4.

Five- and seven-category indicators and the observed univariate distributions make continuous-item ML a pragmatic approximation, not a guarantee of multivariate normality. No ordinal-estimator or robust ordinal-fit analysis was performed; conclusions about measurement fit therefore remain conditional on the continuous-item model. Cronbach's alpha was calculated from item covariances. Omega total was calculated for each raw-score scale from its congeneric loadings, factor variance, and residual variances; omega hierarchical was not estimated. Composite reliability used standardized loadings, and AVE was their mean squared value. Formulae and additional decimal places in S3/S4 explain why different coefficients can round alike (Hayes and Coutts, 2020).

Discriminant validity was described using HTMT based on absolute item correlations, with 5,000 participant-resampling percentile confidence intervals (seed 20260908), and comparison of latent factor correlations with square roots of AVE (Fornell and Larcker, 1981; Henseler et al., 2015). Harman's first-component variance and a one-factor CFA were restricted diagnostics. They cannot rule out common-source bias; no marker variable, temporally separated measurement, or independently measured method factor was available (Podsakoff et al., 2003).

The hypothesis models used observed item-mean scores and ordinary least squares corresponding to PROCESS Model 14 (Hayes, 2022). This transparent composite-score analysis preserves the administered scale metrics but does not correct structural coefficients for measurement error and is not a latent moderated mediation model. Let WM_c = WM – mean(WM) and PP_c = PP – mean(PP). The equations were WM = i_M + a·IL + e_M and VB = i_Y + c′·IL + b1·WM_c + b_2_·PP_c + b3·WM_c·PP_c + e_Y. A separate VB = i_T + c·IL + e_T equation estimated the total association for H1.

H2 used a; H3 used b1 at mean PP; H4 used a·b1 at mean PP; H5 used b3 and ΔR^2^ relative to the outcome model without the product; and H6 used a·b3. Conditional indirect associations were a·[b1 + b3·(PP – mean(PP))]. Percentile bootstrap intervals used 5,000 participant-level resamples (seed 20260806), holding the original sample's raw PP reference values fixed across resamples. Reference values were its mean and mean ± one SD. Conventional coefficient and simple-slope intervals used Student's t distribution with the model residual degrees of freedom. Johnson–Neyman boundaries used the same covariance matrix and t critical value.

The interaction effect size was ΔR^2^/(1 – R^2^_full). Supplementary standardized coefficients express changes in SD units; the standardized interaction equals b3·SD(WM)·SD(PP)/SD(VB). Mean centering used original item-mean metrics and did not harmonize the five- and seven-point scales. A positive indirect product is a conditional statistical association, not evidence of longitudinal or causal mediation (Maxwell and Cole, 2007).

The primary model included the prespecified focal variables to provide a transparent unadjusted association model. This choice does not establish absence of confounding. Sensitivity models included clinical tenure in months, dummy-coded education and department type, and hospital fixed effects (Becker, 2005; Bernerth and Aguinis, 2016). Age was described rather than entered alongside tenure. HC3 intervals assessed heteroskedasticity (Long and Ervin, 2000). Further checks excluded cases with Cook's distance > 4/N or at least ten consecutive identical numeric responses, and omitted each hospital in turn. Diagnostic thresholds were not treated as grounds for deleting otherwise eligible cases. Hospital fixed effects and leave-one-site-out checks do not correct within-unit dependence. Department type is not a unique nursing-unit identifier, and five hospital clusters do not support reliable routine cluster-asymptotic inference.

## 4 Results

### 4.1 Sample and data quality

The retained sample comprised 464 nurses, including 429 women (92.5%). Mean age was 24.25 years (*SD* = 1.98; range 19–31), and mean clinical tenure was 33.56 months (*SD* = 15.82; range 6–60). Hospital contributions ranged from 89 to 98 participants (Table 2). All scale means were exactly reproduced from the item responses; no out-of-range or missing focal-item value was found. The largest Cook's distance in the primary VB equation was 0.03166, and 17 cases exceeded 4/464. These cases remained in the primary analysis.

**Table 2:** Participant characteristics (*N* = 464).

Characteristic	Category or statistic	*n*	%
Gender	Female	429	92.5
Gender	Male	35	7.5
Age, years	*M* = 24.25, *SD* = 1.98, range = 19–31	-	-
Education	Technical secondary school or below	17	3.7
Education	Junior college	218	47.0
Education	Bachelor's degree	223	48.1
Education	Master's degree or above	6	1.3
Clinical tenure, months	*M* = 33.56, *SD* = 15.82, range = 6–60	-	-
Department	Internal medicine	115	24.8
Department	Surgery	129	27.8
Department	Emergency department	57	12.3
Department	Intensive care unit	47	10.1
Department	Operating room	40	8.6
Department	Obstetrics and gynecology	26	5.6
Department	Pediatrics	22	4.7
Department	Outpatient department	14	3.0
Department	Other	14	3.0
Hospital site	Hospital 1	95	20.5
Hospital site	Hospital 2	98	21.1
Hospital site	Hospital 3	92	19.8
Hospital site	Hospital 4	89	19.2
Hospital site	Hospital 5	90	19.4
Hospital level	Grade A tertiary hospital	464	100.0

Hospital labels are anonymized sites. Department type identifies a category, not a unique nursing unit.

### 4.2 Measurement results

The reproduced four-factor CFA yielded χ^2^*(*428) = 475.98, *p* = 0.054, CFI = 0.994, TLI = 0.994, RMSEA = 0.016, and SRMR = 0.040. Merged-factor models fitted less well (Table 3). Standardized loadings ranged from 0.740 to 0.816; alpha and omega total ranged from approximately 0.899 to 0.936, and AVE from 0.581 to 0.608 (Table 4). Alpha, omega, and composite reliability are not identical at full precision; their apparent equality at three decimals reflects rounding and similar within-scale loadings.

**Table 3:** Prespecified alternative measurement models.

Model	*χ^2^*	*df*	CFI	TLI	RMSEA	SRMR
Four-factor	475.98	428	0.994	0.994	0.016	0.040
Three-factor	1276.30	431	0.902	0.894	0.065	0.063
Two-factor	2478.90	433	0.763	0.746	0.101	0.098
One-factor	5347.91	434	0.431	0.391	0.156	0.206

ML treats items as continuous; χ^2^ uses Wishart (*N* − 1) scaling. SRMR uses off-diagonal standardized covariance residuals. Three factors merge WM/VB; two factors merge IL/WM/VB; one factor merges all items. No residual covariance or cross-loading is freely estimated.

**Table 4:** Main-sample measurement reliability and convergent validity.

Scale	Loading range	Alpha	Omega total	CR	AVE
IL	0.740–0.804	0.926	0.926	0.926	0.581
WM	0.742–0.816	0.903	0.903	0.903	0.608
VB	0.753–0.803	0.899	0.899	0.899	0.597
PP	0.740–0.804	0.936	0.936	0.936	0.594

Omega total refers to each raw-score scale. CR uses standardized loadings. Equality after rounding does not imply identical calculations; six-decimal values, formulas, and all 31 loadings are provided in S3/S4.

HTMT values ranged from 0.082 to 0.572, with all bootstrap upper limits below 0.65 (Table 5). Latent factor correlations and square roots of AVE are reported separately in S4, rather than conflating composite Pearson correlations with latent correlations. The first unrotated principal component accounted for 29.32% of item variance. This and the poor one-factor fit indicate that a single common factor does not reproduce the item structure, but do not establish that common-source bias is absent.

**Table 5A:** Descriptives, correlations, and HTMT.

Panel A. Original-metric item means and Pearson correlations						
Scale	M	SD	IL	WM	VB	PP
IL	3.485	0.792	-			
WM	3.513	0.820	0.480^***^	-		
VB	3.486	1.027	0.425^***^	0.516^***^	-	
PP	3.471	0.998	−0.068	−0.081	−0.042	—

^***^p < 0.001. IL/WM, five-point formats; VB/PP, seven-point formats. Raw means should not be compared as construct levels. Pearson intervals and *p*-values are in S4.

**Table 5B:** HTMT estimates with 95% percentile bootstrap intervals.

Panel B. HTMT estimates with 95% percentile bootstrap intervals				
Scale	IL	WM	VB	PP
IL	—			
WM	0.524 [0.445, 0.596]	—		
VB	0.466 [0.386, 0.540]	0.572 [0.494, 0.642]	—	
PP	0.091 [0.074, 0.174]	0.102 [0.077, 0.179]	0.082 [0.069, 0.159]	—

HTMT uses absolute item correlations; 5,000 participant bootstrap resamples, seed 20260908. Latent factor correlations and square roots of AVE are reported separately in S4.

### 4.3 Descriptive associations

IL, WM, and VB were positively correlated (*r* = 0.425–0.516, all *p* < 0.001). PP had small negative correlations with IL (*r* = −0.068), WM (*r* = −0.081), and VB (*r* = −0.042); none was statistically significant. For PP–VB, the 95% Pearson correlation interval included zero (see S4). PP had mean 3.471, SD 0.998, and observed range 1.0–6.5 on its seven-point response format. These values describe this sample and do not by themselves establish restriction of range relative to a population.

### 4.4 Regression equations and conditional associations

Table 6 separates the WM, full VB, and total-association equations, including every coefficient and intercept. IL was associated with WM (*a* = 0.4962, 95% CI [0.4132, 0.5792]) and with VB in the total-association model (*c* = 0.5513, 95% CI [0.4441, 0.6585]). The direct coefficient in the full VB equation was *c*′ = 0.2974, 95% CI [0.1857, 0.4091]. These estimates were consistent with H1 and H2, while leaving their causal interpretation unresolved.

**Table 6A:** Complete regression equations.

Panel A. Outcome: work meaningfulness (WM)					
Predictor	*b*	*SE*	*t*	*p*	95% CI
Intercept	1.7838	0.1509	11.823	< 0.001	[1.4873, 2.0803]
IL	0.4962	0.0422	11.753	< 0.001	[0.4132, 0.5792]

*N* = 464; *R*^2^ = 0.2302; adjusted *R*^2^ = 0.2285; *F*(1, 462) = 138.137, *p* < 0.001.

**Table 6B:** Regression predicting constructive voice: full conditional model.

Panel B. Outcome: constructive voice (VB), full conditional model					
Predictor	b	SE	t	p	95% CI
Intercept	2.4595	0.2021	12.170	< 0.001	[2.0624, 2.8567]
IL	0.2974	0.0569	5.231	< 0.001	[0.1857, 0.4091]
WM_c	0.5043	0.0550	9.162	< 0.001	[0.3961, 0.6124]
PP_c	0.0030	0.0398	0.076	0.939	[−0.0751, 0.0812]
WM_c × PP_c	0.1500	0.0508	2.952	0.003	[0.0501, 0.2498]

*N* = 464; *R*^2^ = 0.3206; adjusted *R*^2^ = 0.3146; *F*(4, 459) = 54.139, *p* < 0.001.

**Table 6C:** Separate total-association regression predicting constructive voice.

Panel C. Separate total-association model, outcome: VB					
Predictor	*b*	*SE*	*t*	*p*	95% CI
Intercept	1.5648	0.1950	8.026	< 0.001	[1.1817, 1.9479]
IL	0.5513	0.0546	10.105	< 0.001	[0.4441, 0.6585]

*N* = 464; *R*^2^ = 0.1810; adjusted *R*^2^ = 0.1792; *F*(1, 462) = 102.115, *p* < 0.001.

IL, WM, VB, and PP denote item means. WM_c and PP_c use full-sample mean centering. Conventional Student-t intervals are reported. The product adds Δ*R*^2^ = 0.0129; Δ*F*(1, 459) = 8.715; *f*^2^ = 0.0190. These are separate association equations, not temporal or causal estimates.

At mean PP, the WM–VB coefficient was 0.5043 (95% CI [0.3961, 0.6124]), consistent with H3. PP's coefficient at mean WM was 0.0030 (95% CI [−0.0751, 0.0812], *p* = 0.939). This is a conditional coefficient, not a test of whether personality is relevant at every WM value. The full VB model explained 32.06% of variance, *F*(4, 459) = 54.139, *p* < 0.001.

The WM × PP coefficient was 0.1500 (95% CI [0.0501, 0.2498], *p* = 0.003). Adding the product explained 1.29 percentage points of additional variance, Δ*F*(1, 459) = 8.715, *p* = 0.003, with *f*^2^ = 0.0190. The standardized interaction coefficient was 0.1194. Thus, the interaction was modest: a one-SD difference in PP was associated with approximately 0.119 additional SD of VB per one-SD difference in WM, holding the other terms fixed.

At raw PP values 2.473, 3.471, and 4.469 (mean – one SD, mean, and mean + one SD), the WM–VB slopes were 0.3546, 0.5043, and 0.6539, respectively, with intervals above zero (Table 7; Figure 2). This supports amplification at these three reference values, consistent with H5. It does not imply that the association exists only among highly proactive nurses, or that it is significant at every observed PP score.

**Table 7A:** Simple slopes and conditional indirect associations.

Panel A. Conditional WM–VB slopes					
PP reference	Raw PP	Slope	SE	95% CI	*p*
Low	2.473	0.3546	0.0758	[0.2057, 0.5036]	< 0.001
Mean	3.471	0.5043	0.0550	[0.3961, 0.6124]	< 0.001
High	4.469	0.6539	0.0738	[0.5088, 0.7990]	< 0.001

**Table 7B:** Conditional indirect associations and index of moderated mediation.

Panel B. Conditional indirect products and index			
Quantity	Estimate	Bootstrap SE	95% bootstrap CI
Indirect at low PP	0.1760	0.0374	[0.1050, 0.2501]
Indirect at mean PP	0.2502	0.0339	[0.1857, 0.3197]
Indirect at high PP	0.3245	0.0455	[0.2379, 0.4174]
Index: a × b3	0.0744	0.0242	[0.0269, 0.1229]

Low/mean/high PP are the sample mean minus one SD, the mean, and the mean plus one SD. H4 uses the mean reference; H6 uses the index. Bootstrap intervals use 5,000 participant resamples, seed 20260806, holding original-sample raw PP reference values fixed. Conventional t-based slope intervals and percentile product intervals are distinct. The Johnson–Neyman region is described in Section 4.4 and S4.

**Figure 2:**
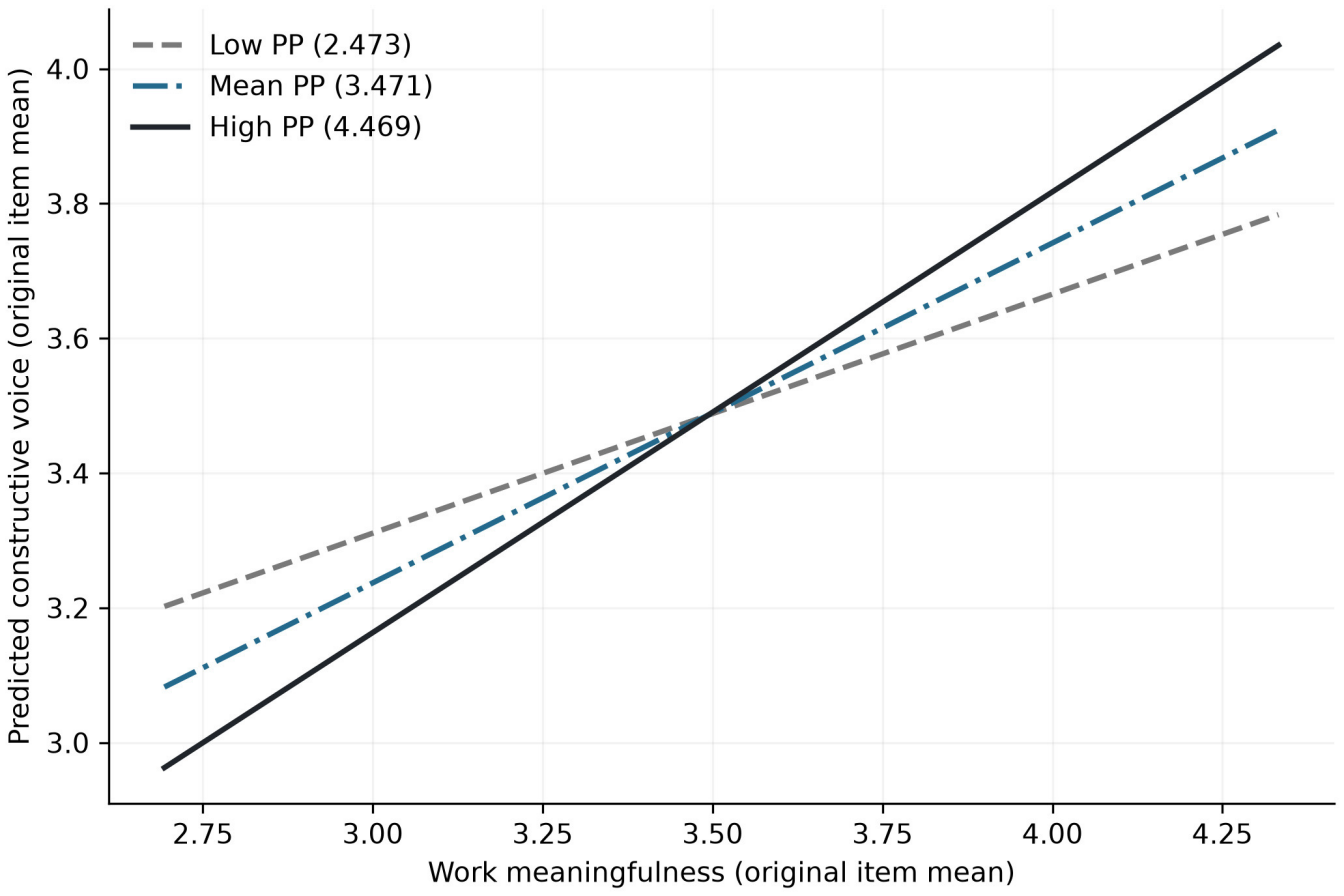
Predicted constructive voice across meaningfulness at three personality reference values. Predictions hold IL at its sample mean. WM spans its sample mean ± one SD; low/mean/high PP also use mean ± one SD and mean. Axes retain original item-mean metrics. Lines describe the fitted regression and are not intervention predictions.

The Johnson–Neyman roots on the raw PP metric were approximately −6.759 and 1.577. Only the second lay within the observed range of 1.0–6.5. Within that range, the positive WM–VB slope reached *p* < 0.05 for PP > 1.577; it was not significant for 1.0 ≤ PP ≤ 1.577. Fourteen nurses (3.0%) were at or below the observed-range boundary and 450 (97.0%) were above it. The negative root was outside both the observed range and the possible scale. These regions are inferential descriptions of a fitted model, not validated clinical cutoffs.

The conditional indirect association at mean PP was 0.2502 (95% bootstrap CI [0.1857, 0.3197]), consistent with H4. At low and high PP it was 0.1760 (95% CI [0.1050, 0.2501]) and 0.3245 (95% CI [0.2379, 0.4174]), respectively. The index of moderated mediation was 0.0744 (95% CI [0.0269, 0.1229]), consistent with H6. Table 7 distinguishes these bootstrap quantities from regression coefficients and simple slopes. The bootstrap intervals use the fixed raw PP reference values specified in Section 3.7.

### 4.5 Sensitivity analyses

HC3 inference retained a positive interaction, and the model with tenure, education, department type, and hospital fixed effects estimated an interaction of 0.1606. Removing the 17 Cook-flagged cases reduced the interaction estimate to 0.1291, compared with 0.1500 in all 464 cases; excluding 15 long-response-run cases yielded 0.1486. Supplementary File S4 reports conventional and HC3 intervals, indirect-association intervals, sample sizes, and leave-one-hospital-out estimates. These checks assess sensitivity to observed cases and site composition; they do not establish robustness to unmeasured confounding, common-source bias, or within-supervisor dependence.

## 5 Discussion

### 5.1 Contribution and interpretation

In this sample, perceived inclusive leadership, meaningfulness, and constructive voice were positively associated, and the meaningfulness–voice association was stronger at higher proactive personality. The positive conditional indirect product is consistent with the proposed model. It does not show that leadership first changes meaningfulness or that meaningfulness subsequently produces voice. Favorable work appraisals, reverse relationships, and unmeasured personal or organizational characteristics remain alternative explanations.

The contribution is a conditional value-of-work account alongside existing safety-focused explanations. Psychological safety addresses whether expression is interpersonally tolerable; meaningfulness addresses why improvement might be worth pursuing. The latter is consistent with leadership–meaningful work–voice research (Chen et al., 2018), and differs in emphasis from nursing research on psychological safety (Lee and Dahinten, 2021). Because psychological safety, respect, and belongingness were not measured, we cannot claim incremental explanatory power over these alternatives or superiority of meaningfulness. Their joint assessment is a necessary next test.

The personality interaction locates a proposed boundary at the meaningfulness–voice association, contrasting with a leadership–reflection-stage specification in Xu et al. (2024). This difference is a proposition about where conditionality may occur, not evidence that the alternative is incorrect. Social information processing theory provides a plausible interpretation of leader cues and appraisals, while trait activation offers a limited interactionist rationale. The present data neither measure cue activation nor warrant claims of a new trait-activation mechanism. The early-career setting adds population-specific evidence, with no test of career-stage differences.

### 5.2 The unexpected personality correlations

The absence of a positive PP–VB zero-order relationship deserves attention because proactive personality and constructive voice both involve initiative. It differs from the positive proactivity–voice account examined by Xie et al. (2014). We checked permissible item ranges, the specified positive scoring direction, recomputed all ten-item means, and reproduced the negative, near-zero correlations. No simple reverse-coding or mean-calculation error was identified. This audit verifies the supplied item coding, not the accuracy of the administered translation or data collection.

Several explanations remain possible. Nurses may express initiative through actions other than upward communication, and workplace constraints could limit whether a general disposition is expressed as voice. Translated items may capture a somewhat different emphasis, and a selected early-career sample could differ from broader employee populations. However, contextual suppression, measurement differences, and restricted range were not directly tested. The observed PP SD and range alone cannot establish restriction, and the small adjusted main coefficient does not establish statistical suppression. These possibilities should be investigated rather than used to explain away the discrepancy.

The positive product term can coexist with a weak marginal correlation because it describes differences in slopes across meaningfulness. It supports a conditional association in this dataset, but does not restore evidence for a general positive PP–VB relationship or make personality a useful screening instrument. Replication with documented translations, multiple sources, and comparison populations is particularly important for this part of the model.

### 5.3 Magnitude and practical implications

The interaction explained an additional 1.29 percentage points of variance (*f*^2^ = 0.0190). Slopes were positive at low, mean, and high PP, so the pattern is principally amplification across commonly used probing values. Although the fitted slope was not significant in the lowest observed PP region, that region contained only 14 participants. Neither the interaction nor its significance boundary establishes a meaningful change in clinical practice, and practical importance cannot be inferred from *p*-values alone.

Three practice considerations follow cautiously from the observed associations and the wider voice literature (Nembhard and Edmondson, 2006; Morrison, 2023). First, managers may consider consistent invitations for suggestions and constructive responses to input. Second, discussing how nursing contributions matter may be worth evaluating alongside efforts to make speaking up safe. Third, accessible opportunities and optional preparation or feedback support can be offered to all nurses with differing comfort in speaking up. These proposals require intervention evaluation; the study did not test reporting platforms, handover redesign, or other implementation programs. Personality scores should not be used for recruitment, exclusion, or allocation of voice opportunities on the basis of these results. No improvement in patient safety or care quality was demonstrated.

### 5.4 Limitations and future research

First, all focal variables were self-reported at one time. The analysis cannot establish temporal sequence or causal mediation, and shared response styles, social desirability, affect, or leader-evaluation concerns could distort associations. Because human resources staff knew participation status and reminded non-respondents, perceived pressure to participate or concerns about evaluation may also have influenced participation and answers, even though these staff reportedly did not view individual responses. Confidentiality protections and the measurement diagnostics do not eliminate these possibilities. Stronger designs should separate measurement occasions and obtain supervisor-, peer-, or independently coded voice measures; a justified marker or method model may complement, but cannot replace, improved design.

Second, selection of five Grade A tertiary hospitals in one city through accessible human resources contacts and purposive recruitment limit generalizability. Eligible-population totals were not counted at the hospitals. Personnel-record screening and a target of 100 invitations per hospital therefore do not establish population-proportional coverage, known inclusion probabilities, or a representative sample. The delivered invitation count is based on investigator confirmation, and the high submission proportion does not eliminate selection or non-response bias. Findings may differ among experienced nurses, other hospital settings, other regions, or non-nursing occupations. Comparison groups and documented probability-based recruitment, where feasible, would better assess those boundaries. The undocumented basis and timing of the completion-time cutoff also limit assessment of selection bias introduced by questionnaire screening.

Third, nurses may share supervisors or nursing units. Hospital fixed effects and leave-one-hospital-out checks assess aspects of site composition, but do not correct correlated residuals within units or supervisors. Department categories do not identify unique units, and five hospitals provide too few clusters for dependable routine cluster-asymptotic standard errors. Leadership-coefficient and indirect-association uncertainty may therefore be understated. Future sampling should record non-identifying unit/supervisor codes and include enough independent clusters for multilevel inference (Lee et al., 2025).

Fourth, preliminary translation, pilot assessment, and CFA do not establish comprehensive cross-cultural validity. All focal items were positively worded, leaving acquiescence concerns (Schriesheim and Hill, 1981). Continuous-item ML approximates ordinal responses, and the composite-score path analysis ignores measurement error. Ordinal measurement models, invariance tests, and latent interaction models would provide informative extensions. The unexpectedly weak PP correlations strengthen the case for independent assessment of item meaning and external validity.

Finally, neither psychological safety nor other competing explanations was included, and demographic adjustment does not remove all confounding. The study assessed general constructive voice rather than observed improvement, incident reporting, or patient outcomes. Longitudinal, multisource, and multilevel studies should compare alternative explanatory pathways and evaluate whether the modest interaction replicates before testing practical interventions.

## 6 Conclusion

Among early-career nurses in five tertiary hospitals in Harbin, the observed pattern was consistent with a positive statistical conditional indirect association between inclusive leadership and constructive voice through work meaningfulness that increased with proactive personality. The interaction was modest and occurred alongside a near-zero personality–voice correlation. The findings support a context-specific conditional association model; they do not establish a leadership intervention pathway or improvements in patient safety or care quality. Longitudinal, multisource, and multilevel research is the next step.

## Data Availability

The datasets presented in this article are not readily available because participant consent and the ethics framework do not authorize unrestricted public release of the de-identified participant-level data. Qualified researchers may request access from the corresponding authors at why78326@163.com or 13069076750@163.com. Access is subject to review and permission by the Ethics Committee and/or responsible institution and may require a data-use agreement. The pilot and main-study datasets are stored separately and were not merged for the primary analyses. Requests to access the datasets should be directed to Huiyu Wang, why78326@163.com; He Zhou, 13069076750@163.com.
