# Supplementary material for: Inclusive leadership and voice behavior among early-career nurses: the conditional roles of work meaningfulness and proactive personality
Source: Front Psychol. 2026 Oct 22;17:1964826. doi: 10.3389/fpsyg.2026.1964826 (PMC13735587; doi:10.3389/fpsyg.2026.1964826)
Supplement: Supplementary file 1 [file Data_Sheet_1.zip › S3_Analysis_Methods.docx]

# Supplementary File S3

## Data checks and reproducible analysis

This file describes the revision-stage reproduction of the manuscript’s measurement and composite-score association analyses. The companion reproduce_revision.py file in this archive is executable and produces aggregate results. No participant-level data are included in this package.

## 1. Inputs and scope

Use the authorized restricted formal and pilot XLSX workbooks, each with an Analysis_Data sheet. Expected sizes are 464 formal and 158 pilot records. The formal restricted file has 60 fields, including 31 item responses and four scale means. It excludes ResponseID, StartTime, EndTime, and IPDuplicateFlag. The dictionary must describe this restricted schema separately from any original platform export.

The files contain retained cases only. The code cannot verify 500 invitations, 476 submissions, 12 exclusions, reminders/incentives, pilot collection dates, consent implementation, or participant nonoverlap. Original collection and cleaning records are needed for those matters. No participant record is deleted in the primary analysis.

## 2. Running the code

Python 3.12.14 was used. Install the versions in requirements.txt in an isolated environment. With the restricted files kept locally, run the following command; substitute authorized local paths:

python reproduce_revision.py --formal "FORMAL.xlsx" --pilot "PILOT.xlsx" --output-dir "local_results"

The executable does not use a network connection or upload data. It outputs statistical_results.json containing aggregate model, measurement, and sensitivity results. Software versions and random seeds are recorded. Small numerical differences across library/platform versions can occur; the supplied aggregate JSON records this revision’s full-precision results.

## 3. Scoring and model equations

IL is the mean of IL_1–IL_9, WM of WM_1–WM_6, VB of VB_1–VB_6, and PP of PP_1–PP_10. All items are positive as specified by the supplied questionnaire/dictionary. IL and WM range 1–5; VB and PP range 1–7. All retained item values and recomputed means were checked. Original scale metrics are preserved.

WM = i_M + a·IL + e_M. VB = i_Y + c′·IL + b₁·WM_c + b₂·PP_c + b₃·WM_c·PP_c + e_Y. A separate VB = i_T + c·IL + e_T equation gives the total association. WM_c and PP_c are centered around original full-sample means. In resampling, fixing those centers is algebraically equivalent to recentering each resample while evaluating the same fixed raw moderator references.

The indirect association at a raw PP value w is a·[b₁ + b₃·(w − mean(PP))]; the index is a·b₃. The 5,000-resample percentile bootstrap uses seed 20260806 and holds the original raw mean and mean ± SD PP reference values fixed. Conventional intervals use the model-specific Student-t distribution. Earlier fixed critical values/normal p-value approximations and sample-varying moderator references have been replaced by these explicit conventions.

## 4. CFA specification and reliability formulas

semopy fits MLW models with a marker loading fixed to one for each factor, mutually correlated factors, zero cross-loadings, and zero residual covariances. No modification-index-driven changes are used. Items are treated as continuous; this is not a robust ordinal estimator. Wishart (N − 1) scaling multiplies the ML discrepancy for χ², and the same scaling is used consistently for the baseline comparison. Fit is not used as proof against common-source bias.

**Four-factor**

F1 =~ IL_1 + IL_2 + IL_3 + IL_4 + IL_5 + IL_6 + IL_7 + IL_8 + IL_9
F2 =~ WM_1 + WM_2 + WM_3 + WM_4 + WM_5 + WM_6
F3 =~ VB_1 + VB_2 + VB_3 + VB_4 + VB_5 + VB_6
F4 =~ PP_1 + PP_2 + PP_3 + PP_4 + PP_5 + PP_6 + PP_7 + PP_8 + PP_9 + PP_10

**Three-factor**

F1 =~ IL_1 + IL_2 + IL_3 + IL_4 + IL_5 + IL_6 + IL_7 + IL_8 + IL_9
F2 =~ WM_1 + WM_2 + WM_3 + WM_4 + WM_5 + WM_6 + VB_1 + VB_2 + VB_3 + VB_4 + VB_5 + VB_6
F3 =~ PP_1 + PP_2 + PP_3 + PP_4 + PP_5 + PP_6 + PP_7 + PP_8 + PP_9 + PP_10

**Two-factor**

F1 =~ IL_1 + IL_2 + IL_3 + IL_4 + IL_5 + IL_6 + IL_7 + IL_8 + IL_9 + WM_1 + WM_2 + WM_3 + WM_4 + WM_5 + WM_6 + VB_1 + VB_2 + VB_3 + VB_4 + VB_5 + VB_6
F2 =~ PP_1 + PP_2 + PP_3 + PP_4 + PP_5 + PP_6 + PP_7 + PP_8 + PP_9 + PP_10

**One-factor**

F1 =~ IL_1 + IL_2 + IL_3 + IL_4 + IL_5 + IL_6 + IL_7 + IL_8 + IL_9 + WM_1 + WM_2 + WM_3 + WM_4 + WM_5 + WM_6 + VB_1 + VB_2 + VB_3 + VB_4 + VB_5 + VB_6 + PP_1 + PP_2 + PP_3 + PP_4 + PP_5 + PP_6 + PP_7 + PP_8 + PP_9 + PP_10

For k items with covariance matrix C, alpha = k/(k − 1) × [1 − trace(C)/sum(C)]. For unstandardized loadings λ, factor variance φ, and diagonal residual variances θ, omega total = (sum λ)²φ / [(sum λ)²φ + sum θ]. For standardized loadings λ*, CR = (sum λ*)² / [(sum λ*)² + sum(1 − λ*²)], and AVE = mean(λ*²). These refer to each unidimensional scale within the correlated-factor model; omega hierarchical is not estimated. SRMR is the square root of the mean squared off-diagonal entries of (S − Σ_hat)/sqrt(diag(S)diag(S)ᵀ).

## 5. Additional measurement checks

HTMT uses the mean absolute between-construct item correlations divided by the geometric mean of the two mean absolute within-construct inter-item correlations. Its 5,000 participant-resample percentile intervals use seed 20260908. Harman’s diagnostic is the largest eigenvalue of the 31-item correlation matrix divided by 31. Neither diagnostic isolates or rules out general common-method variance.

Pilot principal axis factoring starts from squared multiple correlations and iterates communalities to a maximum change below 10⁻⁸, retaining four factors. Promax rotation uses power 4. Parallel analysis was added during revision: 2,000 independent within-column permutations preserve item category distributions; observed correlation eigenvalues are compared with position-specific 95th-percentile reference eigenvalues. The reduced-matrix PAF eigenvalues and full correlation-matrix eigenvalues are different quantities. Factor signs and column order in the pattern matrix are arbitrary.

## 6. Sensitivity analysis and inference limits

Conventional and HC3 Student-t intervals are reported. Adjusted equations include clinical tenure, education, department type, and hospital indicators; each categorical reference is the smallest numeric code. The code also excludes Cook’s-distance flags (>4/N), excludes response runs of at least ten identical values across the fixed IL/WM/VB/PP item order, and omits each hospital in turn. Cases remain in the primary analysis. Department type does not identify a unique unit; none of these checks corrects within-supervisor dependence. Bootstrap resampling is at the participant level, so it likewise assumes independent records.

The Johnson–Neyman roots solve (b₁ + b₃w_c)² = t²[V(b₁) + 2w_cCov(b₁,b₃) + w_c²V(b₃)], using the full outcome-model covariance and 459 residual degrees of freedom. Results are reported in centered and raw PP units and restricted to the actual observed range. The standardized interaction equals b₃·SD(WM)·SD(PP)/SD(VB), rather than standardizing the product as a standalone observed variable.

The sample-size calculation is a noncentral-F check of the stated f² = 0.05, one-tested/four-total-predictor assumptions. It does not constitute an ordinal-CFA, indirect-effect, or clustered-design power analysis. CFA examines measurement; OLS estimates observed-composite associations and ignores structural measurement error. Cross-sectional indirect products are not causal effects.
