# Supplementary material for: Inclusive leadership and voice behavior among early-career nurses: the conditional roles of work meaningfulness and proactive personality
Source: Front Psychol. 2026 Oct 22;17:1964826. doi: 10.3389/fpsyg.2026.1964826 (PMC13735587; doi:10.3389/fpsyg.2026.1964826)
Supplement: Supplementary file 1 [file Data_Sheet_1.zip › S4_Additional_Results.docx]

# Supplementary File S4

## Additional measurement and analysis results

All tables below were recomputed from the restricted formal (N = 464) and pilot (N = 158) item datasets. Coefficients retain original item-mean metrics unless identified as standardized. Raw participant records are not included. These are revision-stage results with the limitations described in the manuscript and S3.

## S4.1 Reliability and measurement model

| **Scale** | **Alpha** | **Omega total** | **CR** | **AVE** |
| --- | --- | --- | --- | --- |
| IL | 0.925831 | 0.925979 | 0.925809 | 0.581136 |
| WM | 0.902942 | 0.903194 | 0.902893 | 0.608010 |
| VB | 0.898802 | 0.898931 | 0.898920 | 0.597221 |
| PP | 0.935816 | 0.935901 | 0.935890 | 0.593619 |

| **Item** | **Standardized loading** |
| --- | --- |
| IL_1 | 0.758890 |
| IL_2 | 0.739839 |
| IL_3 | 0.759255 |
| IL_4 | 0.749920 |
| IL_5 | 0.784703 |
| IL_6 | 0.761048 |
| IL_7 | 0.759968 |
| IL_8 | 0.803783 |
| IL_9 | 0.741302 |
| WM_1 | 0.767931 |
| WM_2 | 0.777495 |
| WM_3 | 0.741656 |
| WM_4 | 0.799371 |
| WM_5 | 0.773445 |
| WM_6 | 0.816443 |
| VB_1 | 0.802983 |
| VB_2 | 0.753437 |
| VB_3 | 0.770960 |
| VB_4 | 0.767555 |
| VB_5 | 0.770982 |
| VB_6 | 0.770028 |
| PP_1 | 0.740200 |
| PP_2 | 0.756958 |
| PP_3 | 0.775028 |
| PP_4 | 0.775409 |
| PP_5 | 0.804495 |
| PP_6 | 0.774072 |
| PP_7 | 0.774590 |
| PP_8 | 0.760432 |
| PP_9 | 0.796452 |
| PP_10 | 0.744578 |

| **Model** | **χ² (N−1)** | **df** | **p** | **CFI** | **TLI** | **RMSEA** | **SRMR** |
| --- | --- | --- | --- | --- | --- | --- | --- |
| Four-factor | 475.978 | 428 | 0.054 | 0.9944 | 0.9940 | 0.0156 | 0.0397 |
| Three-factor | 1276.304 | 431 | < 0.001 | 0.9022 | 0.8945 | 0.0651 | 0.0627 |
| Two-factor | 2478.902 | 433 | < 0.001 | 0.7632 | 0.7457 | 0.1010 | 0.0983 |
| One-factor | 5347.908 | 434 | < 0.001 | 0.4313 | 0.3907 | 0.1564 | 0.2058 |

MLW discrepancy scaled by N−1; no cross-loadings or correlated residuals. Off-diagonal SRMR convention. No ordinal sensitivity estimator was fitted.

**Latent factor correlations (off diagonal) and square roots of AVE (diagonal)**

| **Factor** | **IL** | **WM** | **VB** | **PP** |
| --- | --- | --- | --- | --- |
| IL | 0.7623 | 0.5300 | 0.4667 | −0.0712 |
| WM | 0.5300 | 0.7798 | 0.5779 | −0.0891 |
| VB | 0.4667 | 0.5779 | 0.7728 | −0.0477 |
| PP | −0.0712 | −0.0891 | −0.0477 | 0.7705 |

Order F1 = IL, F2 = WM, F3 = VB, F4 = PP. Latent correlations, not composite Pearson correlations, are used for this AVE comparison.

| **Pair** | **HTMT** | **95% bootstrap CI** |
| --- | --- | --- |
| IL–WM | 0.5236 | [0.4448, 0.5962] |
| IL–VB | 0.4660 | [0.3863, 0.5403] |
| IL–PP | 0.0912 | [0.0739, 0.1736] |
| WM–VB | 0.5718 | [0.4940, 0.6422] |
| WM–PP | 0.1023 | [0.0766, 0.1790] |
| VB–PP | 0.0817 | [0.0691, 0.1586] |

5,000 participant resamples; seed 20260908. First-component variance = 29.3226%. Method bias remains unresolved.

## S4.2 Pilot factor retention and pattern coefficients

| **Component** | **Observed eigenvalue** | **Permutation 95th percentile** | **Retained** |
| --- | --- | --- | --- |
| 1 | 7.2485 | 2.0789 | Yes |
| 2 | 5.4958 | 1.8979 | Yes |
| 3 | 2.8825 | 1.7813 | Yes |
| 4 | 2.0781 | 1.6872 | Yes |
| 5 | 0.9628 | 1.6015 | No |
| 6 | 0.8027 | 1.5272 | No |
| 7 | 0.7743 | 1.4578 | No |
| 8 | 0.7450 | 1.3933 | No |

Parallel analysis was added during revision; four factors had originally been prespecified. Correlation-matrix values are shown here; PAF reduced-matrix eigenvalues were 6.767, 5.008, 2.402, and 1.602.

| **Item** | **Factor 1** | **Factor 2** | **Factor 3** | **Factor 4** |
| --- | --- | --- | --- | --- |
| IL_1 | −0.7643 | 0.0039 | 0.0390 | 0.0100 |
| IL_2 | −0.7253 | −0.0217 | −0.0242 | −0.0247 |
| IL_3 | −0.6415 | −0.0241 | −0.0287 | −0.0400 |
| IL_4 | −0.7433 | −0.0250 | 0.0321 | −0.0595 |
| IL_5 | −0.6803 | −0.0336 | 0.0365 | 0.0834 |
| IL_6 | −0.6847 | 0.0395 | 0.0137 | 0.0336 |
| IL_7 | −0.6269 | 0.1099 | 0.0154 | 0.1107 |
| IL_8 | −0.7280 | −0.1160 | −0.0956 | 0.0378 |
| IL_9 | −0.6016 | 0.0619 | −0.0253 | −0.0126 |
| WM_1 | 0.1018 | 0.0004 | −0.1301 | 0.5952 |
| WM_2 | −0.0398 | 0.1330 | 0.0964 | 0.7159 |
| WM_3 | −0.0769 | −0.0087 | −0.1511 | 0.6495 |
| WM_4 | −0.0028 | −0.0556 | 0.0961 | 0.8261 |
| WM_5 | −0.0993 | −0.0884 | 0.0703 | 0.6619 |
| WM_6 | −0.0189 | 0.0156 | −0.1004 | 0.6232 |
| VB_1 | 0.0509 | 0.0544 | −0.8355 | 0.0215 |
| VB_2 | −0.0261 | 0.0554 | −0.7424 | −0.1378 |
| VB_3 | −0.0587 | 0.0056 | −0.6107 | 0.0452 |
| VB_4 | 0.0223 | −0.0537 | −0.6535 | 0.1465 |
| VB_5 | 0.0320 | −0.0206 | −0.6712 | 0.1555 |
| VB_6 | −0.0268 | −0.0290 | −0.7497 | −0.0886 |
| PP_1 | −0.0908 | 0.6864 | 0.0018 | −0.0986 |
| PP_2 | 0.0796 | 0.6182 | 0.0846 | 0.1493 |
| PP_3 | −0.0174 | 0.7796 | −0.0627 | −0.0832 |
| PP_4 | 0.0109 | 0.7400 | −0.0538 | 0.0055 |
| PP_5 | 0.0330 | 0.6801 | 0.1170 | 0.0377 |
| PP_6 | −0.0481 | 0.6701 | 0.0323 | 0.0255 |
| PP_7 | −0.0622 | 0.6817 | −0.0482 | −0.2170 |
| PP_8 | 0.1058 | 0.7063 | −0.1485 | 0.0423 |
| PP_9 | −0.0442 | 0.7434 | −0.0379 | −0.0746 |
| PP_10 | 0.0380 | 0.6909 | 0.0880 | 0.2369 |

Promax power 4, factor signs/order arbitrary. This independently reproduced pattern replaces the original rounded pattern summaries. KMO = 0.884916; Bartlett χ²(465) = 2322.226618; N = 158. The strongest loading for each item defines its primary column.

## S4.3 Descriptives and Pearson correlations

| **Scale** | **M** | **SD** | **Min** | **Max** | **Skewness** | **Excess kurtosis** |
| --- | --- | --- | --- | --- | --- | --- |
| IL | 3.4847 | 0.7925 | 1.3333 | 5.0000 | −0.2237 | −0.4818 |
| WM | 3.5129 | 0.8196 | 1.1667 | 5.0000 | −0.3490 | −0.3424 |
| VB | 3.4860 | 1.0269 | 1.1667 | 6.3333 | −0.0276 | −0.3222 |
| PP | 3.4713 | 0.9980 | 1.0000 | 6.5000 | 0.0249 | −0.0887 |

| **Pair** | **r** | **p** | **95% Fisher interval** |
| --- | --- | --- | --- |
| IL–WM | 0.4798 | < 0.001 | [0.4065, 0.5469] |
| IL–VB | 0.4255 | < 0.001 | [0.3479, 0.4972] |
| IL–PP | −0.0685 | 0.141 | [−0.1585, 0.0227] |
| WM–VB | 0.5163 | < 0.001 | [0.4462, 0.5801] |
| WM–PP | −0.0810 | 0.081 | [−0.1708, 0.0101] |
| VB–PP | −0.0421 | 0.365 | [−0.1326, 0.0491] |

Item-level skewness range: [−0.374, 0.214]; excess kurtosis range: [−0.579, −0.078]. Full-score means reproduce the provided scale columns exactly.

## S4.4 Primary and covariate-adjusted regression equations

**Primary WM equation**

| **Predictor** | **b** | **SE** | **t** | **p** | **95% CI** |
| --- | --- | --- | --- | --- | --- |
| Intercept | 1.7838 | 0.1509 | 11.823 | < 0.001 | [1.4873, 2.0803] |
| IL | 0.4962 | 0.0422 | 11.753 | < 0.001 | [0.4132, 0.5792] |

N = 464; R² = 0.2302; adjusted R² = 0.2285; F(1, 462) = 138.137, p < 0.001.

**Primary VB equation**

| **Predictor** | **b** | **SE** | **t** | **p** | **95% CI** |
| --- | --- | --- | --- | --- | --- |
| Intercept | 2.4595 | 0.2021 | 12.170 | < 0.001 | [2.0624, 2.8567] |
| IL | 0.2974 | 0.0569 | 5.231 | < 0.001 | [0.1857, 0.4091] |
| WMc | 0.5043 | 0.0550 | 9.162 | < 0.001 | [0.3961, 0.6124] |
| PPc | 0.0030 | 0.0398 | 0.076 | 0.939 | [−0.0751, 0.0812] |
| WMxPP | 0.1500 | 0.0508 | 2.952 | 0.003 | [0.0501, 0.2498] |

N = 464; R² = 0.3206; adjusted R² = 0.3146; F(4, 459) = 54.139, p < 0.001.

**Separate total-association equation**

| **Predictor** | **b** | **SE** | **t** | **p** | **95% CI** |
| --- | --- | --- | --- | --- | --- |
| Intercept | 1.5648 | 0.1950 | 8.026 | < 0.001 | [1.1817, 1.9479] |
| IL | 0.5513 | 0.0546 | 10.105 | < 0.001 | [0.4441, 0.6585] |

N = 464; R² = 0.1810; adjusted R² = 0.1792; F(1, 462) = 102.115, p < 0.001.

**Covariate-adjusted WM equation**

| **Predictor** | **b** | **SE** | **t** | **p** | **95% CI** |
| --- | --- | --- | --- | --- | --- |
| Intercept | 1.8115 | 0.2644 | 6.851 | < 0.001 | [1.2918, 2.3311] |
| IL | 0.5113 | 0.0428 | 11.952 | < 0.001 | [0.4272, 0.5954] |
| ClinicalTenureMonth | 0.0015 | 0.0022 | 0.691 | 0.490 | [−0.0028, 0.0058] |
| Education_2 | −0.1053 | 0.1826 | −0.577 | 0.564 | [−0.4642, 0.2536] |
| Education_3 | 0.0210 | 0.1830 | 0.115 | 0.909 | [−0.3385, 0.3806] |
| Education_4 | −0.0715 | 0.3476 | −0.206 | 0.837 | [−0.7547, 0.6116] |
| DepartmentType_2 | −0.0312 | 0.0937 | −0.332 | 0.740 | [−0.2154, 0.1531] |
| DepartmentType_3 | −0.0769 | 0.1180 | −0.652 | 0.515 | [−0.3087, 0.1550] |
| DepartmentType_4 | −0.1486 | 0.1256 | −1.184 | 0.237 | [−0.3954, 0.0982] |
| DepartmentType_5 | −0.2886 | 0.1331 | −2.168 | 0.031 | [−0.5502, −0.0270] |
| DepartmentType_6 | −0.0248 | 0.1574 | −0.158 | 0.875 | [−0.3341, 0.2845] |
| DepartmentType_7 | −0.4492 | 0.1689 | −2.660 | 0.008 | [−0.7811, −0.1174] |
| DepartmentType_8 | −0.3300 | 0.2060 | −1.602 | 0.110 | [−0.7350, 0.0749] |
| DepartmentType_9 | −0.1606 | 0.2049 | −0.784 | 0.433 | [−0.5632, 0.2420] |
| HospitalID_2 | −0.0263 | 0.1055 | −0.249 | 0.803 | [−0.2336, 0.1810] |
| HospitalID_3 | 0.0781 | 0.1061 | 0.736 | 0.462 | [−0.1304, 0.2865] |
| HospitalID_4 | 0.0071 | 0.1071 | 0.067 | 0.947 | [−0.2033, 0.2176] |
| HospitalID_5 | −0.0320 | 0.1074 | −0.298 | 0.766 | [−0.2431, 0.1791] |

N = 464; R² = 0.2577; adjusted R² = 0.2295; F(17, 446) = 9.110, p < 0.001.

Education, department type, and HospitalID use categorical indicators; omitted reference is code 1. ClinicalTenureMonth is continuous. See the coding companion for category labels. These adjustments do not correct unit dependence or eliminate confounding.

**Covariate-adjusted VB equation**

| **Predictor** | **b** | **SE** | **t** | **p** | **95% CI** |
| --- | --- | --- | --- | --- | --- |
| Intercept | 1.9604 | 0.3252 | 6.029 | < 0.001 | [1.3214, 2.5995] |
| IL | 0.3052 | 0.0577 | 5.292 | < 0.001 | [0.1918, 0.4185] |
| WMc | 0.4977 | 0.0556 | 8.949 | < 0.001 | [0.3884, 0.6070] |
| PPc | 0.0190 | 0.0400 | 0.474 | 0.636 | [−0.0596, 0.0975] |
| WMxPP | 0.1606 | 0.0516 | 3.116 | 0.002 | [0.0593, 0.2619] |
| ClinicalTenureMonth | 0.0039 | 0.0026 | 1.534 | 0.126 | [−0.0011, 0.0089] |
| Education_2 | 0.2807 | 0.2145 | 1.309 | 0.191 | [−0.1409, 0.7022] |
| Education_3 | 0.3141 | 0.2147 | 1.463 | 0.144 | [−0.1078, 0.7360] |
| Education_4 | −0.4758 | 0.4076 | −1.167 | 0.244 | [−1.2769, 0.3253] |
| DepartmentType_2 | 0.0129 | 0.1103 | 0.117 | 0.907 | [−0.2039, 0.2296] |
| DepartmentType_3 | 0.2294 | 0.1388 | 1.653 | 0.099 | [−0.0434, 0.5021] |
| DepartmentType_4 | 0.0483 | 0.1481 | 0.326 | 0.744 | [−0.2427, 0.3393] |
| DepartmentType_5 | −0.0435 | 0.1575 | −0.276 | 0.783 | [−0.3531, 0.2661] |
| DepartmentType_6 | 0.0388 | 0.1859 | 0.209 | 0.835 | [−0.3265, 0.4042] |
| DepartmentType_7 | 0.0050 | 0.2000 | 0.025 | 0.980 | [−0.3881, 0.3981] |
| DepartmentType_8 | −0.2947 | 0.2430 | −1.213 | 0.226 | [−0.7723, 0.1828] |
| DepartmentType_9 | 0.0318 | 0.2411 | 0.132 | 0.895 | [−0.4421, 0.5057] |
| HospitalID_2 | 0.1886 | 0.1247 | 1.512 | 0.131 | [−0.0565, 0.4336] |
| HospitalID_3 | −0.0311 | 0.1247 | −0.249 | 0.803 | [−0.2762, 0.2141] |
| HospitalID_4 | 0.1507 | 0.1257 | 1.199 | 0.231 | [−0.0964, 0.3978] |
| HospitalID_5 | −0.1305 | 0.1261 | −1.034 | 0.301 | [−0.3784, 0.1174] |

N = 464; R² = 0.3545; adjusted R² = 0.3253; F(20, 443) = 12.163, p < 0.001.

Education, department type, and HospitalID use categorical indicators; omitted reference is code 1. ClinicalTenureMonth is continuous. See the coding companion for category labels. These adjustments do not correct unit dependence or eliminate confounding.

## S4.5 HC3 and case/site sensitivity

| **Model** | **N** | **Interaction b** | **Conventional CI** | **HC3 CI** |
| --- | --- | --- | --- | --- |
| Primary | 464 | 0.1500 | [0.0501, 0.2498] | [0.0583, 0.2416] |
| Adjusted | 464 | 0.1606 | [0.0593, 0.2619] | [0.0639, 0.2574] |
| Cook_exclusion | 447 | 0.1291 | [0.0298, 0.2284] | [0.0497, 0.2084] |
| Longrun_exclusion | 449 | 0.1486 | [0.0440, 0.2532] | [0.0514, 0.2458] |
| Leave_hospital_1_out | 369 | 0.1343 | [0.0230, 0.2455] | [0.0289, 0.2397] |
| Leave_hospital_2_out | 366 | 0.1309 | [0.0100, 0.2517] | [0.0199, 0.2418] |
| Leave_hospital_3_out | 372 | 0.1528 | [0.0428, 0.2629] | [0.0532, 0.2525] |
| Leave_hospital_4_out | 375 | 0.1622 | [0.0519, 0.2725] | [0.0612, 0.2632] |
| Leave_hospital_5_out | 374 | 0.1629 | [0.0543, 0.2715] | [0.0615, 0.2643] |

All primary cases retained. Cook exclusion removes 17/464 = 3.7%; long-run exclusion removes 15/464 = 3.2%. Site sensitivity omits each hospital rather than attempting unreliable five-cluster asymptotic inference.

**Primary WM coefficients with HC3 inference**

| **Predictor** | **b** | **HC3 SE** | **HC3 p** | **HC3 95% CI** |
| --- | --- | --- | --- | --- |
| Intercept | 1.7838 | 0.1535 | < 0.001 | [1.4822, 2.0853] |
| IL | 0.4962 | 0.0423 | < 0.001 | [0.4130, 0.5794] |

**Primary VB coefficients with HC3 inference**

| **Predictor** | **b** | **HC3 SE** | **HC3 p** | **HC3 95% CI** |
| --- | --- | --- | --- | --- |
| Intercept | 2.4595 | 0.1995 | < 0.001 | [2.0676, 2.8515] |
| IL | 0.2974 | 0.0554 | < 0.001 | [0.1885, 0.4063] |
| WMc | 0.5043 | 0.0547 | < 0.001 | [0.3967, 0.6118] |
| PPc | 0.0030 | 0.0376 | 0.936 | [−0.0708, 0.0769] |
| WMxPP | 0.1500 | 0.0466 | 0.001 | [0.0583, 0.2416] |

**Adjusted WM coefficients with HC3 inference**

| **Predictor** | **b** | **HC3 SE** | **HC3 p** | **HC3 95% CI** |
| --- | --- | --- | --- | --- |
| Intercept | 1.8115 | 0.2691 | < 0.001 | [1.2826, 2.3404] |
| IL | 0.5113 | 0.0426 | < 0.001 | [0.4276, 0.5950] |
| ClinicalTenureMonth | 0.0015 | 0.0021 | 0.482 | [−0.0027, 0.0057] |
| Education_2 | −0.1053 | 0.1945 | 0.588 | [−0.4875, 0.2769] |
| Education_3 | 0.0210 | 0.1933 | 0.913 | [−0.3589, 0.4010] |
| Education_4 | −0.0715 | 0.3875 | 0.854 | [−0.8331, 0.6900] |
| DepartmentType_2 | −0.0312 | 0.1005 | 0.757 | [−0.2286, 0.1663] |
| DepartmentType_3 | −0.0769 | 0.1127 | 0.496 | [−0.2984, 0.1447] |
| DepartmentType_4 | −0.1486 | 0.1196 | 0.214 | [−0.3836, 0.0863] |
| DepartmentType_5 | −0.2886 | 0.1356 | 0.034 | [−0.5552, −0.0220] |
| DepartmentType_6 | −0.0248 | 0.1488 | 0.868 | [−0.3173, 0.2677] |
| DepartmentType_7 | −0.4492 | 0.1732 | 0.010 | [−0.7897, −0.1088] |
| DepartmentType_8 | −0.3300 | 0.1869 | 0.078 | [−0.6973, 0.0373] |
| DepartmentType_9 | −0.1606 | 0.2250 | 0.476 | [−0.6028, 0.2815] |
| HospitalID_2 | −0.0263 | 0.1074 | 0.807 | [−0.2373, 0.1847] |
| HospitalID_3 | 0.0781 | 0.1075 | 0.468 | [−0.1333, 0.2894] |
| HospitalID_4 | 0.0071 | 0.1074 | 0.947 | [−0.2040, 0.2182] |
| HospitalID_5 | −0.0320 | 0.1067 | 0.764 | [−0.2417, 0.1777] |

**Adjusted VB coefficients with HC3 inference**

| **Predictor** | **b** | **HC3 SE** | **HC3 p** | **HC3 95% CI** |
| --- | --- | --- | --- | --- |
| Intercept | 1.9604 | 0.2948 | < 0.001 | [1.3811, 2.5398] |
| IL | 0.3052 | 0.0578 | < 0.001 | [0.1916, 0.4187] |
| WMc | 0.4977 | 0.0552 | < 0.001 | [0.3893, 0.6061] |
| PPc | 0.0190 | 0.0388 | 0.626 | [−0.0574, 0.0953] |
| WMxPP | 0.1606 | 0.0492 | 0.001 | [0.0639, 0.2574] |
| ClinicalTenureMonth | 0.0039 | 0.0025 | 0.120 | [−0.0010, 0.0089] |
| Education_2 | 0.2807 | 0.1799 | 0.119 | [−0.0729, 0.6342] |
| Education_3 | 0.3141 | 0.1801 | 0.082 | [−0.0399, 0.6681] |
| Education_4 | −0.4758 | 0.5626 | 0.398 | [−1.5814, 0.6298] |
| DepartmentType_2 | 0.0129 | 0.1146 | 0.911 | [−0.2123, 0.2380] |
| DepartmentType_3 | 0.2294 | 0.1333 | 0.086 | [−0.0326, 0.4914] |
| DepartmentType_4 | 0.0483 | 0.1763 | 0.784 | [−0.2982, 0.3948] |
| DepartmentType_5 | −0.0435 | 0.1546 | 0.779 | [−0.3472, 0.2603] |
| DepartmentType_6 | 0.0388 | 0.1957 | 0.843 | [−0.3459, 0.4235] |
| DepartmentType_7 | 0.0050 | 0.2011 | 0.980 | [−0.3902, 0.4001] |
| DepartmentType_8 | −0.2947 | 0.2484 | 0.236 | [−0.7829, 0.1935] |
| DepartmentType_9 | 0.0318 | 0.2708 | 0.907 | [−0.5005, 0.5640] |
| HospitalID_2 | 0.1886 | 0.1288 | 0.144 | [−0.0645, 0.4416] |
| HospitalID_3 | −0.0311 | 0.1301 | 0.811 | [−0.2868, 0.2247] |
| HospitalID_4 | 0.1507 | 0.1306 | 0.249 | [−0.1059, 0.4073] |
| HospitalID_5 | −0.1305 | 0.1290 | 0.312 | [−0.3841, 0.1231] |

**Cook exclusion — conditional indirect products**

| **Quantity** | **Estimate** | **Bootstrap SE** | **95% bootstrap CI** |
| --- | --- | --- | --- |
| Low | 0.1996 | 0.0336 | [0.1367, 0.2676] |
| Mean | 0.2622 | 0.0321 | [0.2014, 0.3261] |
| High | 0.3248 | 0.0422 | [0.2437, 0.4094] |
| Index | 0.0627 | 0.0207 | [0.0227, 0.1028] |

**Longrun exclusion — conditional indirect products**

| **Quantity** | **Estimate** | **Bootstrap SE** | **95% bootstrap CI** |
| --- | --- | --- | --- |
| Low | 0.1716 | 0.0374 | [0.0983, 0.2447] |
| Mean | 0.2433 | 0.0348 | [0.1773, 0.3136] |
| High | 0.3149 | 0.0476 | [0.2262, 0.4105] |
| Index | 0.0718 | 0.0249 | [0.0242, 0.1229] |

Sensitivity bootstrap probes hold the ORIGINAL full-sample raw PP values fixed, even when a subset has a different mean. Resampling assumes independent participants. Adjusted-model bootstrap products are not claimed here.

## S4.6 Interaction probes and standardized aids

| **PP reference** | **Raw PP** | **WM–VB slope** | **95% conventional CI** |
| --- | --- | --- | --- |
| Low | 2.4734 | 0.3546 | [0.2057, 0.5036] |
| Mean | 3.4713 | 0.5043 | [0.3961, 0.6124] |
| High | 4.4693 | 0.6539 | [0.5088, 0.7990] |

| **Quantity** | **Estimate** | **Bootstrap SE** | **95% bootstrap CI** |
| --- | --- | --- | --- |
| Low | 0.1760 | 0.0374 | [0.1050, 0.2501] |
| Mean | 0.2502 | 0.0339 | [0.1857, 0.3197] |
| High | 0.3245 | 0.0455 | [0.2379, 0.4174] |
| Index | 0.0744 | 0.0242 | [0.0269, 0.1229] |

| **Standardized quantity** | **Value** | **Definition** |
| --- | --- | --- |
| interaction | 0.119442 | b₃ × SD(WM) × SD(PP) / SD(VB) |
| wm_at_mean_pp | 0.402507 | b₁ × SD(WM) / SD(VB) |
| IL_direct | 0.229514 | c′ × SD(IL) / SD(VB) |

ΔR² = 0.012900; f² = 0.018986. The interaction’s standardized coefficient is not the coefficient obtained by standardizing the product variable separately.

| **Quantity** | **Value** |
| --- | --- |
| Centered roots | [−10.230133, −1.893963] |
| Raw PP roots | [−6.758797, 1.577373] |
| Observed PP range | [1.000, 6.500] |
| t critical, df = 459 | 1.96514575 |
| Observed nonsignificant region | 1.0 ≤ PP ≤ 1.577373 (14 participants) |
| Observed positive significant region | 1.577373 < PP ≤ 6.5 (450 participants) |

Regions use the conventional model covariance matrix; p = 0.05 at the fitted boundary. The other root is outside the possible scale. These values are not clinical screening thresholds.

Figure S4. Pointwise model-based intervals across the observed PP range. The lower interval limit crosses zero at approximately 1.577; the out-of-scale root is intentionally omitted.

## S4.7 Planning-assumption sensitivity

| **N** | **Power under f² = 0.05** |
| --- | --- |
| 159 | 0.800103 |
| 450 | 0.997225 |
| 464 | 0.997790 |

One tested predictor, four total predictors, α = 0.05, noncentral-F calculation. This is an explanatory check of stated assumptions, not observed-effect post-hoc power or evidence of an originally simulated CFA/indirect-effect design.
