# Supplementary material for: Inclusive leadership and voice behavior among early-career nurses: the conditional roles of work meaningfulness and proactive personality
Source: Front Psychol. 2026 Oct 22;17:1964826. doi: 10.3389/fpsyg.2026.1964826 (PMC13735587; doi:10.3389/fpsyg.2026.1964826)
Supplement: Supplementary file 2 [file Table_2.docx]

Supplementary File S1

# Measure sources scoring and adaptation

This supplement describes the four measures used in the Chinese questionnaire. It reports their sources, item counts, response formats, scoring, and adaptations. Complete Chinese and English item wording is not reproduced. No statement of unrestricted permission to use, translate, or reproduce the instruments is made.

| **Measure** | **Source** | **Items** | **Administered format** |
| --- | --- | --- | --- |
| Inclusive leadership (IL) | Carmeli et al. (2010) | 9 | Five points; supervisor or nurse manager referent |
| Work meaningfulness (WM) | May et al. (2004) | 6 | Five points; nursing work context |
| Constructive voice (VB) | Van Dyne and LePine (1998) | 6 | Seven points; nurse self-report |
| Proactive personality (PP) | Seibert et al. (1999); based on Bateman and Crant (1993) | 10 | Seven points; shortened measure |

# Response formats and scoring

IL and WM used five response categories from 1 (strongly disagree) to 5 (strongly agree), with 3 indicating neutral or uncertain. VB and PP used seven categories from 1 (strongly disagree) to 7 (strongly agree), with 4 indicating neutral or uncertain. The reported formats describe this study’s administered questionnaire.

All 31 focal items were positively worded and none was reverse scored. Each score is the arithmetic mean of its constituent items, retaining full precision. IL and WM scores range from 1 to 5; VB and PP scores range from 1 to 7. Higher means indicate more of the corresponding construct. Means from the five- and seven-point formats should not be compared as equivalent construct levels.

The questionnaire scoring instructions required at least 80% of a construct’s items to be answered before calculating its mean. The 464 retained formal cases and 158 valid pilot cases had complete responses to all 31 focal items, so each reported mean used the full item set. The attention-check item was excluded from all four scale scores. Variable coding is documented in S2.

# Translation and adaptation

The English-language measures were translated into Chinese and adapted to the nursing setting. The study report describes forward translation, synthesis, independent back-translation, expert review, and cognitive interviewing, as detailed in manuscript Section 3.4. IL referred to the nurse’s immediate supervisor or nurse manager; WM referred to nursing work; VB was administered as nurse self-report; PP used the ten-item shortened form cited above. These contextual adaptations do not constitute new instrument development.

The pilot ran from 8 to 30 January 2026. No items, response options, or survey procedures were changed after the pilot. The investigators confirmed that the Chinese items and options in the original full-questionnaire study record match those administered. Pilot and main-study analyses provide preliminary measurement evidence, not comprehensive validation of a standardized Chinese version.

# Original measure sources

Bateman, T. S., & Crant, J. M. (1993). The proactive component of organizational behavior: A measure and correlates. Journal of Organizational Behavior, 14(2), 103–118. https://doi.org/10.1002/job.4030140202

Carmeli, A., Reiter-Palmon, R., & Ziv, E. (2010). Inclusive leadership and employee involvement in creative tasks in the workplace: The mediating role of psychological safety. Creativity Research Journal, 22(3), 250–260. https://doi.org/10.1080/10400419.2010.504654

May, D. R., Gilson, R. L., & Harter, L. M. (2004). The psychological conditions of meaningfulness, safety and availability and the engagement of the human spirit at work. Journal of Occupational and Organizational Psychology, 77(1), 11–37. https://doi.org/10.1348/096317904322915892

Seibert, S. E., Crant, J. M., & Kraimer, M. L. (1999). Proactive personality and career success. Journal of Applied Psychology, 84(3), 416–427. https://doi.org/10.1037/0021-9010.84.3.416

Van Dyne, L., & LePine, J. A. (1998). Helping and voice extra-role behaviors: Evidence of construct and predictive validity. Academy of Management Journal, 41(1), 108–119. https://doi.org/10.5465/256902
